# Impact of corporate governance and CEO remuneration on bank capitalization strategies and payout decision in income shocks period

**DOI:** 10.3389/fpsyg.2022.901868

**Published:** 2022-08-29

**Authors:** Hangqin Xiang, Erum Shaikh, Muhammad Nawaz Tunio, Waqas Ahmad Watto, Yiqing Lyu

**Affiliations:** ^1^Faculty of Engineering, The University of Sydney, Sydney, NSW, Australia; ^2^Department of Business Administration, Shaheed Benazir Bhutto University, Nawabshah, Pakistan; ^3^Department of Management Science, Mohammad Ali Jinnah University, Karachi, Pakistan; ^4^Bahauddin Zakariya University, Multan, Pakistan; ^5^School of Economics, Sichuan University, Chengdu, China

**Keywords:** corporate governance, bank capitalization, capital structure, dividend payout, banking sector

## Abstract

The purpose of this research is to explore corporate governance and CEO remuneration with banks capitalization strategies and payout policy within the Pakistani banking context. Data were obtained from the financial statements of scheduled banks listed on the Pakistan stock exchange from 2005 to 2020. The findings of the research study revealed that corporate governance mechanisms that promote the bank's shareholders' interests are linked to low capitalization strategies. The size of the board of directors has a significant impact on the capitalization of banks. Banks' capitalization techniques are also adversely correlated with effective board size. The shareholder benefits from low capitalization. Corporate governance is positively related with banking sector instability, as seen by this negative correlation. Bank capitalization strategies have a significant impact on CEO remuneration. In the event of an income shock, dividend payout is essential. Banking sector payout policies are negatively related with corporate governance. In the event of a negative income shock, financial institutions reduce dividends. As a result, it has been argued that effective corporate governance benefits shareholders by reducing capitalization tactics and limiting financial institutions' aggressive payouts.

**JEL classification:**

G21, G30, G32, G35.

## Introduction

The financial sector of every country is the lifeblood of the economy. The modern trades and commerce are financed by these financial institutions. The strength of financial institutions signifies the strength of the economy (De Andres et al., 2005; Hussain et al., 2018). As the national income increases, people are encouraged to increase savings and deposits. Modern and large financial institutions facilitate companies to acquire and supervise more money efficiently, which enhances economic growth. A bank-oriented financial system has a bigger effect on growth as compared to a financial system that is market-oriented (Tadesse, 2002; Fase and Abma, 2003). Therefore, financial sector development is very important for the economic development (Andersen and Tarp, 2003).

A financial institution having insufficient capital is a failing financial institution (Anginer et al., 2016). So, that financial institution's likelihood of failure is determined with the capitalization strategies. Moreover, financial institutions use two capitalization strategies. First, financial institutions' level or amount of capital when the business is in normal conditions. High capitalization has a direct and positive effect on the performance of the stock market during the financial crisis period (Beltratti and Stulz, 2012; Beck et al., 2013). Berger and Bouwman (2013) found that the financial institutions maintained a higher level of capital before the crises, so the probability of survival increased under unfavorable conditions, and also found that high capitalized bank stock performance is higher as compared to the low capitalized bank.

Academic research and the 2009 financial crisis have recognized the significance of corporate governance with respect to banks' stability (Mehran et al., 2011). However, the results suggest that corporate governance improved financial institutions' stability long before the start of financial crises. Laeven and Levine (2009) show that board composition has an effect on banks' financial stability. Furthermore, it has been suggested that manager ownership and bank risk-taking have a direct effect. Cole and White (2012) examined the causes of USA commercial bank failure in 2009 by using the logit regression model on all the accounting-based constructs. The conclusion of this research study is that banks having a higher quality of earnings, high liquidity, and more capital significantly decreased their probability of failure. Berger and Bouwman (2013) explained the relationship between the bank capital and performance of the USA banking sector. The findings of this study explain that capital of the banks is the predominate factor for the survival of banking corporations during market and bank crises as well as during normal time periods. The prediction related to insolvency is very important within the banking sector context (Zywicki and Adamson, 2009).

The second strategy relates to decreasing the payout ratio to the shareholder of the banks after observing any income shock in order to maintain a level of capital. Conservative policy makers in the banks decide to increase the issue of shares and cut the dividend as well as share repurchase after facing the loss (Anginer et al., 2016). The banks operating worldwide paid dividends in the financial crises period in 2008 and, before the end of these crises, risk was increased (Acharya and Richardson, 2009). Financial institutions, especially shareholders aiming to maximize their share value, might favor capitalization strategies which are risky. Before crises, banks taking more risk are favored by intestinal investors (Hong et al., 2012). This study empirically investigates the different aspects of corporate governance and the relationship with capitalization strategies of the financial institutions within the context of Pakistan.

Kim and Lu (2011) examined the association between risk-taking by corporate and CEO ownership within the presence of external governance. Findings suggest that corporate governance enhances the risk-taking of the corporation. DeYoung and Torna (2013) explain the relationship between the risky business policy decision and CEO compensation in the USA, and found that CEOs of large financial institutions were aggressive to risk-taking. Different research studies examined the effects of several corporate governance mechanisms' impacts on banks. Several theoretical and empirical papers consider how corporate governance affects banks (Beltratti and Stulz, 2009; Tanda, 2015). The reason shareholders may be less interested in aligning the compensation packages to the management is debt holder interest and deposit insurance. This study tries to fill the gap by analyzing the effect of corporate governance and risky capitalization strategies. This study considers the following aspects of corporate governance: board size, CEO and board chair separation, board independence, and board size effectiveness.

Corporate governance practice in financial institutions is different from non-financial firms (Becht et al., 2011). Several studies consider corporate governance practices in the financial sector of Pakistan with performance (Burki and Ahmad, 2010), dividend policy (Fahim et al., 2015), corporate social responsibilities (Sharif and Rashid, 2014), the evolution of corporate governance in Islamic and conventional banks (Halkias et al., 2013), growth (Ghauri et al., 2012), capital structure (Ahmed Sheikh and Wang, 2012), financial sector liberalization (Di Patti and Hardy, 2005), ownership and financing (Javid and Iqbal, 2010), customer satisfaction (Jamal and Naser, 2003), services quality (Muhammad Awan et al., 2011), and customer loyalty (Mohsan et al., 2011) but does not consider corporate governance and bank capitalization strategies in Pakistan. Therefore, this study was attempted to fill this gap.

## Literature review

### Theoretical background

American literature founded agency theory in the early 1970's. In this decade, one theory also promoted was corporate governance, which is embedded in agency theory. Two relationships exist in the agency theory; one is the principle and the other is the agent. The relationships of principle usually refer to the owner (shareholder) and the relationship of agent usually refers to the board of directors. According to Alchian and Demsetz (1972), the agency theory is rooted in the theory of the firm, and then elaborates its dimension. Famous economist Smith (1976) included the ground rules of agency theory in his book. According to Smith, a person can never expect from the other person who manages his money either for his benefit or not. According to Bratton (2000) the expansion of this theory originates only in the 1970's, yet (Berle and Means, 1932) highlighted the idea of extrication from the control of government since the 1930's. The study of all these authors describes that deviation between ownership and control have possible conflict among investors (shareholders) and executives (management). Agency theory describes that the principle (shareholders) is expecting that the agent (directors) will guide and formulate decisions for the benefit of them, and for those who were given permission to make decisions. Alternatively, the decisions of directors (agent) do not support the interests of the principle. Berle and Means (1932) first described the concept of conflict of interests among the managers and owners, followed by Ross (1973) and Smith (1976), and the work of these authors was expanded by Jensen and Meckling (1976). Agency theory directs decision-making to require executives to align objectives with those of the investor for the purpose of making the best use of company worth that could not be affected by the competing objectives of managers. Hence, this research study uses the Agency Theory to develop the theoretical model.

### Board size and capital structure

Board size means how many directors in the board. A large board has different directors with different knowledge backgrounds, which consequently resolves any agency issue (Ahmed Haji, 2013). Within the presence of the large board, the monitoring power has been enhanced and the innovation has been improved due to the exchange of ideas (Esa and Anum Mohd Ghazali, 2012; Giannarakis, 2014). Esa and Anum Mohd Ghazali (2012) conducted the study in the Malaysian context. The purpose of the study was to investigate the relationship between corporate governance and corporate social responsibilities. The results suggest that CSR enhances corporate governance mechanisms.

Coles et al. (2008) explored the relationship between the board structure and firm performance in Taiwan. The results suggest that the small board size has a positive effect on Taiwan firms as compared to the large board size. Kiel and Nicholson (2003) conducted the research to find the relationship between corporate performance and firm value in the Australian context. The results suggest that the board size and firm size have a positive influence on the firm value.

In the banking sector, this question is widely analyzed. Many studies have been done to check the relationship between the variety measures of the firm performance and board size (return on assets or return on equity) for the financial company. There have been numerous studies which elaborate that banks' performance and size of the board are positively correlated, whereas the non-financial firms are totally distinct. According to Adams and Mehran (2012) from 1965 to 1999, the firm performance in large US banks and board size shows positive significance. The same conclusion was reached by Aebi et al. (2012) in regard to the financial crisis of US banks. On the basis of the above literature, the following hypothesis is formulated.

**H1: Larger boards may be associated with lower or higher bank capitalization, while boards of intermediate size are associated with the lowest bank capitalization**.

### CEO/board chair separation capital structure

Firms' financing decisions must be affected by CEO duality (where the CEO and board chair are the same). A two-tier structure of leadership is where the CEO position and the board of directors chair are not held by the same person. Fama and Jensen (1983) are the first who give the justification and identify the management decision, and they suggested that management has a right to set off and execute the proposals for a firm's expenditure and have a right to monitor those proposals. The CEO exerts their power by not allowing the controlling authority to make a decision, since the board is not under the control of the CEO.

Furthermore, they endorse this decision. If the decision and management in under the one control it does not generate the positive signals (Shaikh, 2019; Nguyen et al., 2021). The board actions are greatly affected by its chair. Board management decisions and controlling decisions must be bargained when the firm CEO and board chair are not one person. A firm should be more effective, and their agency issue must be less, if the chair and CEO persons hold different positions in the firm. Fosberg (2004) said that firms that have a two-tier structure deploy their debt effectively and efficiently in capital structure and describe that the above-cited statement is vice versa if the firm has no optimal position. Debt/equity ratio is higher in two-tier structures, but it cannot be statistically significant.

### Board independence and capital structure

Board independence refers to the number of outside (independent) directors or non-executive directors considered as independent directors. The main task of the non-executive directors is to minimize the agency problem between the principle and the agent. The relationship between corporate governance and firm value has been found to be positive (Cornett et al., 2007; Dahya et al., 2008; Maini and Aggarwal, 2009). According to De Andres and Vallelado (2008), the firm value improved due to the presence of non-executive directors by using their power to monitor the directors and give advice. Some studies exert that there is no relationship between non-executive directors and firm value (Yermack, 1996; Bhagat et al., 2015).

Past studies related to an independent board also made comparisons between the financial and non-financial institutions' board composition. When the comparison has been made, the results highlighted the difference between the financial and non-financial firms' board independence. The proportion of non-executive directors in the content of the United States is between 70 and 80. (Baillie et al., 2002; Adams and Mehran, 2003; Belkhir, 2009), as compared to the non-executive director in the other sectors or non-financial sectors who fall between 60 and 70 (Adams and Mehran, 2003; Bhagat et al., 2015). The on-average results with respect to the non-executive directors also hold true in the setting of other European countries.

De Andres et al. (2012) conducted research on the ownership concentration and the ratio of the non-executive directors and found that the banks having headquarters in civil law administered countries have a higher independent director ratio as compared to other countries where the ownership concentration is low. According to the findings of Minton et al. (2014), board independence and firm performance were negatively associated with each other.

According to the stewardship theory, a manager should have the following characteristics in different countries: they should be trustworthy, have a collectivistic mind, and should be a reorganizational risk taker who will also take care of the shareholders objectives (Klettner, 2021). Stewardship theory also suggests that due to a better understanding regarding the business, the insider made the best decision as compared to the outsiders, and consequently, the wealth of the company is increased. So, previous findings suggest that the independence of the director and firm performance results will always be mixed. Consequently, the board size structure characteristic has a direct and positive influence on the performance of the company (Memon et al., 2019; Govindarajo et al., 2021; Klettner, 2021). According to the suggestion of the resource-based theory, decision-making had been improved due to more board independence, having the knowledge related to the different industries (Barney, 1991; Adams and Mehran, 2012). Since it stimulates the best governance, the results should be the growth in the firm performance (Butler et al., 2013). By using the data of 25 non-financial Canadian firms (Adams and Mehran, 2012; Shaikh et al., 2021, 2022,a), different results were attained. On the basis of the above literature, the following hypothesis is formulated.

**H2: Banks with more independent boards, and with boards not chaired by the CEO, have lower capitalization**.

### CEO compensation and capital structure

When the executive is granted stock options, risk-taking behavior of the manager can become enhanced because of greater returns based on the volatility of the stock having a positive impact on the option's value (Haugen and Senbet, 1978; Smith and Stulz, 1985; Shaikh et al., 2019, 2022). Consequently, the compensation based on the equity does not lead to increased risk-taking behavior by the manager since there is no impact on their value. Nguyen et al. (2021) stated that compensation granted to the manager based on money discourages the risky behavior of the manager; by applying the equaling approach related to certainty, it is concluded that the firm leverage choice also has an influence on the composition of the managers.

Armenia et al. (2021) found that there is a causality relationship between delta and vega. By applying the simulation approach, the portfolio of the CEOs consisting of delta and vega asserts the increasing trend in the case of a riskier firm. This increase in delta and vega leads the corporation policies to enhance the firm risk. Consequently, Aggarwal and Samwick (1999) show that the value of the delta has been decreased due to the higher risk. These findings conform with the study of principle and agent framework. As the risk has been increased, the cost of the capital also increased to provide the incentives based on the equity. However, the endogeneity problem arises when interpreting the findings as regression analysis has been applied. Even the simulation analysis does not overcome the endogeneity problem (Coles and Pivnenko, 2005; Tunio et al., 2021; Shaikh et al., 2022,b).

The compensation based on the equity has led the manager toward risk aversion. From this manager are choices to take optimal risk (Sharma et al., 2021). But the results related to the compensation based on the equity and managerial behavior related to the risk is the endogeneity problem; this problem has been raised when the independent variable has been related to error terms, and the interpretation of the results has been creating problems. The policy related to the investment effects on the manager in a different way, and the interpretation of the results on the basis of separate risk is consolidated. Different studies considered the different variables but, in this paper, we consider the risk of the firm itself as a variable. Based on the above literature, the following hypothesis is formulated.

**H3: While the relationship between overall executive compensation and bank capitalization can be ambiguous, higher risk incentives embedded in executive compensation should be negatively related to bank capitalization**.

### Corporate governance and dividend payout

Lee et al. (2021) suggest that optimum payout policy is obsessed with the prerequisite to distributing the firm's free cash flow. They suggest a life-cycle theory that is the combination of the Jensen (1993) agency theory with development in the firm's investment opportunity set of the type debated. This theory explains that, in the presence of a set of opportunities, firms optimally modify dividends plans at different times. The theory forecasts that, in their initial years, a firm's investment opportunities are higher than their own generated capita, that is why they pay few dividends. In advanced years, to keep the free cash flows from being wasted, firms decided to pay out excess funds in the form of dividends due to an increase in internal funds rather than investment opportunities. Depending upon this life cycle, Lee et al. (2021) found that the tendency to pay dividends is significantly correlated with the ratio of retained earnings to total equity, as it was their substitution for the firm's lifecycle stage.

Kanojia and Bhatia (2021) exert that the quality of the corporate governance and dividend payout is positively associated. As the corporate governance practices have been improved, the payout policy of the firm also increased. Davis and Marquis (2021) found that a strong corporate governance mechanism has been important because the rights of the shareholders or owners are being observed by the other authority and dividend payout has been enhanced. Waris et al. (2021) also found the same results related to governance and payout using control variables such as size, growth, and profitably. According to Widodo et al. (2021) within the presence of the restriction imposed by the company on outsourcing financing, the payout ratios of such types of companies are lower. The firm owner participation in the board of directors enhances the dividend payout. When owners are included in the board, there is a higher rate of dividend. If the managers of the firm are the shareholders, they have an influence on the dividend policy as compared to the minority shareholders. Based on the above literature, the following hypothesis is formulated.

**H4: Corporate governance and executive compensation that are associated with lower bank capitalization are also associated with continued payouts to shareholders after major negative income shocks**.

## Methodology

The purpose of this study is to find out the relationship between executive compensation and corporate governance mechanisms on the capitalization strategies and payout of the financial sector of Pakistan. The population of this study is the financial institutions that are listed on the Pakistan stock exchange. Only conventional banks have been selected for this study. The data set for this analysis was collected from the financial sector of Pakistan from years 2005 to 2020. Only the banks have been selected in this data set analysis, since their structure of the financial statement is different from the non-financial sector. State bank scheduled banks are a total number of 33. Four banks that are specialized financial institutions are not included in the analysis. Four foreign banks whose date of incorporation and working in Pakistan was not started from 2005 were also not included. Five Islamic banks were not included. All remaining banks were included in the analysis. Finally, 20 financial institutions were observed, and the remaining institutions were dropped for lack of data availability within the time of 15 years.

This study used the balance panel data; panel data means observing multiple banks over the multiple periods from 2005 to 2020. The panel data is suitable for examining the dynamic change; the sample size significance also increased. EViews version 9 is used for the data analysis. The relationship between executive compensation and corporate governance mechanism on the capitalization strategies and payout of the financial sector of Pakistan — this study used a fixed effect model. In panel data, the same observations have been taken over different time periods, so there is a chance of cross-sectional effects on each observed firm or group of firms. Different techniques are used to deal with this problem, but the most important econometric techniques are random as well as a fixed effect. This empirical investigation employed the Hausman (1978) test to determine that either the fixed effect model best explained, or random effect model enlightened our estimation. So, on the value of Hausman (1978), the fixed effect model was used.

### Econometric models

Equation (1)


$$\begin{array}{l} CAP_{it}=a_{0}+\beta_{1}{\text{BI}}_{it_{1}}+\beta_{2}{\text{CD}}_{it_{1}}+\beta_{3}{\text{BS}}_{it_{1}}+\beta_{4}{\text{BSE}}_{it_{1}}\\ +\beta_{5}LngAssets_{it_{1}}+\beta_{6}ROA_{it_{1}}+\beta_{7}OS_{it_{1}}+\varepsilon_{it}\\ CAP_{it}=a_{0}+\beta_{1}{\text{REM}}_{it_{1}}+\beta_{2}LngAssets_{it_{1}}+\beta_{3}ROA_{it_{1}}\\ +\beta_{4}OS_{it_{1}}+\;\varepsilon_{it}. \end{array}$$

In the equation, CAP represents the bank capitalization strategies, BI board independence, CD CEO duality role, BS board size, BSE board size effectiveness, Assets natural logarithm of total assets, ROA return on assets, OS ownership concentration, and REM CEO total annual remuneration.

Equation (2)


$$\begin{array}{l} \text{Payout}{=a}_{0}+\beta_{1}{\text{BI}}_{it_{1}}+\beta_{2}CD_{it_{1}}+\beta_{3}BS_{it_{1}}+\beta_{4}BSE_{it_{1}}\\ ++\beta_{5}IC_{it_{1}}+\varepsilon_{it}\\ \text{payout}=a_{0}+\beta_{1}{\text{REM}}_{it_{1}}+\beta_{2}LngAssets_{it_{1}}+\beta_{3}ROA_{it_{1}}\\ +\beta_{4}OS_{it_{1}}+\varepsilon_{it} \end{array}$$

In the equation, payout means the bank's decision is to pay a dividend. This is measured with two variables (DR dividend to total assets, DI dummy of a dividend equal to one if the dividend is paid otherwise zero), where BI means board independence, CD CEO duality role, BS board size, BSE board size effectiveness, IC income shock, REM CEO total annual remuneration, LngAssets natural logarithm of total assets, ROA return on assets, and OS ownership concentration.

## Results

### Descriptive statistics

Table 1 provides the descriptive statistic summary of the dependent variables with respect to independent variables. Dependent variables are capitalization strategies (market value, tangible equity, common equity, tier1, and total capital) and payout policies (dividend which is equal to 1 when a dividend is paid and otherwise zero, i.e, dividend to asset ratio) of the banking sector, while independent variables are corporate governance (board independence, board size. Board size effectiveness) and CEO compensation schemes (total annual compensation includes, house rent, utilities, managerial services fee, gratuity scheme, medical allowance, etc.).

**Table 1:** Descriptive statistics.

**Variable**	**Obs**	**Mean**	**Std. Dev**.	**Min**	**Max**
MV	200	0.1255125	0.0950034	0.0125	0.5479
TCR	200	0.0964075	0.0811334	−0.1407	0.5173
TER	200	0.104555	0.0803588	−0.0248	0.5186
TR	200	0.14266	0.1058315	0.0028	0.6412
TC	200	0.1633755	0.1014315	0.0173	0.6543
BI	200	0.252958	0.2302381	0	0.875
BS	200	8.675	1.530059	4	13
BSE	200	2.485	0.5207021	1	3
NED	200	0.5644685	0.2755589	0	0.9167
REM	200	4.62282	0.4127601	3.5248	5.8686
DI	200	0.62	0.4866045	0	1
DR	200	0.009359	0.0340037	0	0.3161
ASSETS	200	8.335483	0.5075401	6.6047	9.346
ROA	200	0.0092105	0.0255861	−0.1037	0.0541
OS	200	0.6	0.4911273	0	1
INSHOK	200	0.16	0.367526	0	1

Independent variables TC total capital ratio has a mean value of 16.33%. It shows that, on average, the Pakistani banks maintain a higher capital ratio. According to law, the total capital ratio must at least be equal to 10%. The mean value of tier 1 is 14.26%, which is greater than the required 7.5%, which means that on average Pakistani banks have more capital to risk-weighted assets ratios than the requirement of law. The total equity ratio mean value is 10.45%, which represent the investment of common shareholders in the banks'capital, and the remaining 90% of capital consists of other stakeholder investment in the banks. Tangible capital ratio (TCR) mean value is 9.5%. The current corporate governance variable board size mean value is 8.6%, the maximum value is 13, and the minimum is 4. The board size has the effect on the organizational decision-making process. The board size effectiveness mean value is 2.86%; its value represents that the board size, on average, is effective in the banking sector.

Low board size does not compete with the interest of the shareholder and high board size is not effective. High board size creates the free rider problem and decision-making problem also occurs. The board independence means that the percentage of outside directors in the board of the company — the more the number of outside directors, the more the interest of the shareholder aligns with the management. The code of corporate governance requires that one-third of the board of the company must be independent. On average, in Pakistani context, 2.5% of its board is independent. CEO remuneration is the annual total salary provided to the CEO of the company. On average, every CEO is annually compensated with the amount of 4.62 million rupees. The payout is measured with two variables; the first one is dividend dummy, the bank pays a dividend or not. On average, 62% of the Pakistani banks paid yearly dividends. Another variable is the dividend to total assets ratio. The amount of dividend which is returned to the shareholder as compared to the total assets of the company. The mean value is 0.9%. A very low amount of dividend is provided to the shareholder as compared to the assets of the banks.

This study used bank specific, three control variables that have been introduced. The first is a log of the total assets of a financial higher value that indicate the large financial institutions. Large financial institutions maintain lower capitalization strategies. The second is the return on assets, which is equal to the bank before tax has been divided by the overall assets of the bank. As financial institutions earn higher profit, their capitalization ratio automatically increases. The third variable is ownership concentration dummies; one (1) is used when only the owner has 10% ownership, otherwise zero. The ownership which is concentrated is related to low capitalization strategies (Li et al., 2021).

The multicollinearity problem has been determined by using the Pearson correlation coefficient among the dependent variables and independent variables. If the correlation coefficient has a value which is greater than 0.08 (Kennedy, 1985) or greater than 0.9 (Tabachnick and Fidell, 1996), then it shall be considered an alarming problem. Table 2 illustrate the correlation coefficient among the dependent variables and independent variables.

**Table 2:** Correlation matrix.

	**MV**	**TCR**	**TER**	**TR**	**TC**	**BI**	**BS**	**BSE**	**REM**	**DIV**	**DR**	**ASSETS**	**ROA**	**OS**	**INSHOK**
MV	1														
TCR	0.181*	1													
TER	0.641**	0.671**	1												
TR	0.531***	0.195***	0.109***	1											
TC	0.619***	0.718***	0.181*	0.245**	1										
BI	0.554**	0.525**	0.381*	0.445**	0.125*	1									
BS	0.534***	0.701***	0.328*	0.345*	0.500***	0.619***	1								
BSE	0.701***	0.419***	0.381*	0.415**	0.540***	0.695***	0.134***	1							
REM	0.445**	0.349*	0.419**	0.612***	0.231*	0.349*	0.345*	0.45**	1						
DIV	0.716***	0.619***	0.349*	0.199***	0.200*	0.199*	0.560***	0.349*	0.328*	1					
DR	0.531***	0.195***	0.109***	0.610***	0.69***	0.123*	0.459***	0.619***	0.419***	0.381*	1				
ASSETS	0.54**	0.054**	0.381*	0.450**	0.612***	0.132*	0.300*	0.349*	0.349*	0.38*	0.45**	1			
ROA	0.591***	0.601***	0.381*	0.405**	0.329*	0.695***	0.235*	0.619***	0.612***	0.132*	0.45**	0.328*	1		
OS	0.620***	0.531***	0.195***	0.109***	0.333*	0.503***	0.349*	0.349*	0.612***	0.132*	0.381*	0.405**	0.381*	1	
INSHOK	0.534**	0.594**	0.349*	0.334*	0.12*	0.243*	0.113*	0.619***	0.349*	0.349*	0.612***	0.132*	0.138*	0.145**	1

The *, **, *** symbol indicates the level of the significance.

Panel A illustrates the relationship between corporate governance and bank capitalization. The board independence variable is regressed on five bank capitalization proxies, along with three bank-specific control variables; assets determine the size of the bank, return on assets defines profitability of the banks, and ownership defines the concentrated ownership of the banks. Four capitalization variables —common equity to total assets (TER), tangible equity to total tangible assets (TCR), tier 1 ratio (TR), total capital (tier1+ tier2) (TC)— based on accounting measures and on the variable are based on the market measure — market value of common equity to book value of common equity ratio (MV). Board independence has a significant and negative relationship with tangible equity at 1% level, common equity at 1% level, and the total capital ratio at 1% level, and tier 1 at 1%. The first hypothesis is accepted on the premise of Pakistan data results. The bank size has a negative and significant impact on bank capitalization strategy at a 1% level. Ownership concentration has a negative and significant association with market value, tier 1 capital, and total capital at 1 and 10% levels, which represent that ownership concentration increased, capitalization strategies decreased, and return on assets has a negative and significant impact on bank capitalization strategies.

In panel B, the board size variable is regressed on five bank capitalization proxies. Board size has a significant and negative relationship with tangible equity at 1% level, common equity at 1% level, the total capital ratio at 1% level, and tier 1 at 1 %. The hypothesis is accepted on the premise of Pakistan data results. Panel C board size effectiveness variable is regressed on five bank capitalization proxies. Board size effectiveness has a significant and negative relationship with tangible equity at 1% level, common equity at 1% level, the total capital ratio at 1% level, and tier 1 at 1 %. The third hypothesis is accepted on the premise of Pakistan data results. In panel D, CEO duality variables are regressed on five bank capitalization proxies. CEO duality has a significant and negative relationship with tangible equity at 1% level, common equity at 1% level, and the total capital ratio at 1% level, and tier 1 at 1 %. In panel E, CEO compensation variable is regressed on five bank capitalization proxies. CEO compensation has a significant and negative relationship with tangible equity at 1% level, common equity at 1% level, the total capital ratio at 1% level, and tier 1 at 1 %. The fourth hypothesis is accepted on the premise of Pakistan data results.

With reference to Table 3, this section examines the effects of CEO compensation and corporate governance on payout decisions of banking institutions while facing negative income shocks. Payout policies refer to the management decisions regarding whether to distribute, and how much of the earnings of the institution are distributed, to the stockholders as a dividend (Okafor and Chijoke-Mgbame, 2011). A dividend is a portion of profit that is distributed to the stock investor of the firm (Woolridge, 1982).

**Table 3:** Corporate governance CEO remuneration impact on bank capitalization strategies.

**Panel A**	**MV(1)**	**TCR(2)**	**TER(3)**	**TR(4)**	**TC(5)**
BI	(−0.278) (0.006)	(−0.411) (0.057)	(−0.063) (0.003)	(−0.028) (0.011)	(−0.047) (0.081)
ASSETS	(−0.075) (0.003)	(−0.088) (0.000)	(−0.084) (0.001)	(−0.122) (0.005)	(−0.109) (0.002)
ROA	(−0.880) (0.003)	(−0.484) (0.035)	(−0.345) (0.105)	(−0.213) (0.001)	(−0.221) (0.003)
OS	(−0.028) (0.044)	(−0.003) (0.100)	(−0.002) (0.096)	(−0.025) (0.070)	(−0.025) (0.067)
Ajd.R SQUARE	0.446	0.462	0.391	0.295	0.570
**Panel B**					
BS	(−0.130) (0.004)	(−0.180) (0.003)	(−0.309) (0.079)	(−0.395) (0.352)	(−0.792) (0.059)
Ajd.R SQUARE	0.346	0.263	0.467	0.525	0.517
**Panel C**					
BSE	(−0.418) (0.012)	(−0.027) (0.024)	(−0.385) (0.050)	(−0.423) (0.009)	(−0.156) (0.107)
Ajd.R SQUARE	0.397	0.310	0.465	0.386	0.484
**Panel D**					
CD	−0.349 (0.005)	−0.612 (0.100)	−0.132 (0.001)	−0.138 (0.010)	−0.145 (0.003)
Ajd.R SQUARE	0.292	0.251	0.397	0.397	0.310
**Panel E**					
REM	322) (0.016)	225) (0.013)	(0.044) (0.000)	(0.568) (0.017)	(0.168) (0.016)
ASSETS	(0.509) (0.000)	(0.727) (0.000)	(0.399) (0.000)	(0.151) (0.000)	(0.490) (0.000)
ROA	(0.856) (0.004)	(0.553) 0.016)	(0.467) (0.037)	(0.228) (0.042)	(0.229) (0.033)
OS	(0.015) (0.042)	(0.009) (0.011)	(0.0147) (0.0144)	(0.0151) (0.079)	(0.014) (0.048)
Ajd.R SQUARE	0.454	0.522	0.301	0.292	0.251

This empirical investigation used two payout proxies; dividend and dividend to total assets ratio. For dividend dummy, either bank pay dividend is equal to one or otherwise zero. The dividend to total assets ratio is calculated using the amount of dividend which is returned to the shareholder as compared to the total assets of the company. Negative income shock is measured as, when the return on assets is negative and <20% of the previous year's return, then the bank is considered to be in negative income shock equal to one, otherwise zero. Every regression includes an interactive term with independent variables for CEO compensation and corporate governance. If the interactive term coefficient value is positive, then it is considered that the dividend is paid even in negative income shock, which represents banks' risky payout strategies.

Panel A illustrates the relationship between corporate governance and bank payout. The board size effectiveness variable is regressed on bank payout policies. Board size effectiveness has a negative and significant relationship with corporate payouts. Banks with effective board size favor not paying dividends in case of incurred negative income shocks. Panel B board independence variable is regressed on bank payout policies. Board independence has a positive and insignificant relationship with corporate payouts. Panel C board size variable is regressed on bank payout policies. Board size has a negative and significant relationship with corporate payouts. Board size in banks favors not paying dividends in case of incurred negative income shocks, and banks having a small board scale back the dividend in case of negative income. In panel D, CEO duality variables are regressed on five bank capitalization proxies. CEO duality has a significant and negative relationship with payout policy. Panel E illustrates CEO compensation and bank payout. CEO compensation variable is regressed on bank payout policies. CEO compensation has a positive and significant relationship with corporate payouts. Banks continue to pay even in the negative income shocks as mentioned in Table 4.

**Table 4:** Corporate governance, CEO remuneration, and banks pay out policy in case of income shocks.

**Panel A**	**DI**	**DR**
INSHOK	(−1.443) (0.000)	(−0.056) (0.006)
BSE	(−0.480) (0.009)	(−0.465) (0.007)
Ajd.R SQUARE	0.392	
**Panel B**		
INSHOK	(−0.083) (0.007)	(−1.352) (0.000)
BI	(−0.018) (0.018)	(−0.814) (0.448)
Ajd.R SQUARE	0.389	
**Panel C**		
INSHOK	(−1.415) (0.000)	(−0.019) (0.853)
BS	(−0.179) (0.008)	(−0.001) (0.002)
Ajd.R SQUARE	0.408	
**Panel D**		
INSHOK	(−0.215) (0.002)	(−0.602) (0.012)
REM	(−0.432) (0.076)	(−0.0205) (0.0524)
Ajd.R SQUARE	0.408	

The fourth hypothesis is accepted.

## Discussion

The findings of this study showed that the results support the first, second, third, and fourth hypotheses, which state that there is a relationship between board independence, board size effectiveness, board size, CEO duality, CEO remuneration with bank capitalization strategies, and payout policy. Board size effectiveness associated with lower capitalization is consistent with the results of these studies. So, based on findings, effective board size favors the shareholder interest by maintaining minimum capitalization and shifting the risk from shareholder to debt holder in the Pakistani context. While in the case of CEO duality, findings also show a significant negative relationship, which is also consistent with the study of Lee and Ko (2022). Board independent non-executive directors and dividend payout ratio are in support of the fourth hypothesis, which states that there is a relationship between independent non-executive directors and dividend payout ratio among the Malaysian sample companies, which these results also support (Subramaniam et al., 2022). Regarding the control variables, the finding of concentrated ownership is also in line with the assertion that there is a relationship between concentrated ownership and dividend payout ratio. The results show that concentrated ownership has a significant positive influence on the firm dividend payout ratio and is consistent with the previous studies (Lahiri, 2022; Seth and Mahenthiran, 2022). This means that dividend payment can be used in mitigating an agency conflict as it can serve as a substitute for shareholders' monitoring. Consequently, large shareholders will have the courage to require high dividend payments for them to reduce the monitoring costs. There is a significant positive relationship between firm size and dividend payout ratio. This means that the larger firms pay higher dividends than smaller firms, and this is consistent with the previous studies (Yu et al., 2021; Benyadi et al., 2022).

## Conclusion

Maximization of stakeholder wealth is a primary purpose of all types of corporations and banks as well. Financing and payout decisions are paramount to achieving this goal. Financing decisions are related to equity or debt source in assets of the institutions, while the payout is related to the distribution of profit to shareholders or providing capital gains to these shareholders by reinvesting earnings into the assets of the banks. When the stock price has been appreciated, selling these stocks results in capital gains being received. However, this empirical investigation includes only capitalization decisions and payout decisions with respect to corporate governance mechanism and CEO remuneration. In the last decades, due to financial collapses in developed countries, good corporate governance requirements have been increased to protect shareholders and control over the banks' management. The impact of these financial collapses on the economy initiated the introduction of several reforms related to the financial market and stock exchange in Pakistan. Corporate governance codes 2002 implemented and required all the listed firms to follow them.

This research investigates the link between corporate governance and CEO remuneration with banks capitalization strategies and payout policy in Pakistani context. Data is gathered from financial statements of scheduled banks listed on the Pakistan stock exchange in order to analyze results from 2005 through 2020. Corporate governance mechanisms that favor the banks' shareholders' interests are linked to low capitalization strategies in this study. Shareholder-friendly corporate governance is defined as a governance system that includes board independence, a medium board size, and Board chair separation duality. The size of the board of directors has a significant impact on the capitalization of financial organizations. Banks' capitalization strategies are also adversely correlated with effective board size. Risk is shifted from bank shareholders to debt holders as a result of corporate governance. The shareholder benefits from low capitalization. Corporate governance is positively related to banking sector instability, as seen by this negative correlation. Corporate governance has a downside in that it increases the banks' risk. This disadvantage is offset by the benefit of effective governance, which has limited managerial underperformance. Bank capitalization strategies have a detrimental impact on CEO remuneration. When a company's capitalization is low, the CEO's remuneration is raised. According to the 2012 Corporate Governance Code, the head of the board and the CEO must be separate people. The chairman of the board must be a non-executive director, and their position in the board's leadership must be non-executive.

The distribution of residual earnings to the financial institution's owner is referred to as a payout decision; in the event of an income shock, the payout is essential. Banks' payout policies are negatively related to corporate governance; in the event of a negative income shock, financial institutions reduce dividends. As a result, it has been argued that effective corporate governance benefits shareholders by reducing capitalization tactics and limiting banks to aggressive payouts.

This study provides implications for the corporations and financial institutions of developing countries to make financial decisions.

## Limitations and future directions

First, this study only covers scheduled banks listed on the Pakistan stock exchange, and the results do not generalize to other jurisdictions such as other developing countries' and Asian countries' stock exchanges. Second, the sample of the study is only listed financial institutions and their results do not generalize to smaller and non-scheduled banks. Third, only four corporate governance variables—board size, board size effectiveness, board independence, and CEO chairmen duality—have been taken for investigation with capitalization strategies. Many other important variables—corporate governance disclosure, board committee, audit committee, corporate ethics, director fee, board meeting—may also have a significant impact on bank capital. Forth, this study considers some bank board characteristics, including board size, board size effectiveness, board independence, and CEO/Board chair duality; but several characteristics of board age, tenure of directorship, education, and gender characters may also explain this relationship.

## Data availability statement

The original contributions presented in the study are included in the article/supplementary material, further inquiries can be directed to the corresponding author.

## Author contributions

HX: conceiving idea and introduction. ES: literature review. MT: methodology. WW: results and discussion. YL: supervision, revision, resources, and proofread. All authors contributed to the article and approved the submitted version.

## Conflict of interest

The authors declare that the research was conducted in the absence of any commercial or financial relationships that could be construed as a potential conflict of interest.

## Publisher's note

All claims expressed in this article are solely those of the authors and do not necessarily represent those of their affiliated organizations, or those of the publisher, the editors and the reviewers. Any product that may be evaluated in this article, or claim that may be made by its manufacturer, is not guaranteed or endorsed by the publisher.
